# Combined EGFR- and notch inhibition display additive inhibitory effect on glioblastoma cell viability and glioblastoma-induced endothelial cell sprouting in vitro

**DOI:** 10.1186/s12935-016-0309-2

**Published:** 2016-04-26

**Authors:** Mikkel Staberg, Signe Regner Michaelsen, Louise Stobbe Olsen, Mette Kjølhede Nedergaard, Mette Villingshøj, Marie-Thérése Stockhausen, Petra Hamerlik, Hans Skovgaard Poulsen

**Affiliations:** Department of Radiation Biology, The Finsen Center, Copenhagen University Hospital, Copenhagen, Denmark; Department of Clinical Physiology, Nuclear Medicine & PET and Cluster for Molecular Imaging, Copenhagen University Hospital and University of Copenhagen, Copenhagen, Denmark; Brain Tumor Biology Group, Danish Cancer Society Research Center, Copenhagen, Denmark

**Keywords:** Glioblastoma, Angiogenesis, Endothelial spheroid sprouting, Notch, EGFR, DAPT, Iressa, Gamma-secretase inhibitor, Tyrosine-kinase inhibitor

## Abstract

**Background:**

For Glioblastoma (GBM) patients, a number of anti-neoplastic strategies using specifically targeting drugs have been tested; however, the effects on survival have been limited. One explanation could be treatment resistance due to redundant signaling pathways, which substantiates the need for combination therapies. In GBM, both the epidermal growth factor receptor (EGFR) and the notch signaling pathways are often deregulated and linked to cellular growth, invasion and angiogenesis. Several studies have confirmed cross-talk and co-dependence of these pathways. Therefore, this study aimed at testing a combination treatment strategy using inhibitors targeting the notch and EGFR pathways.

**Methods:**

For evaluation of cell viability a standard MTT assay was used. Western blotting (WB) and Q-RT-PCR were employed in order to assess the protein- and mRNA expression levels, respectively. In order to determine angiogenic processes, we used an endothelial spheroid sprouting assay. For assessment of secreted VEGF from GBM cells we performed a VEGF-quantikine ELISA.

**Results:**

GBM cells were confirmed to express EGFR and Notch and to have the capacity to induce endothelial cell sprouting. Inhibition of EGFR and Notch signaling was achieved using either Iressa (gefitinib) or the gamma-secretase inhibitor DAPT. Our data showed that DAPT combined with Iressa treatment displayed increased inhibitory effect on cell viability and abrogated expression and activation of major pro-survival pathways. Similarly, the combinational treatment significantly increased abrogation of GBM-induced endothelial cell sprouting suggesting reduced GBM angiogenesis.

**Conclusion:**

This study finds that simultaneous targeting of notch and EGFR signaling leads to enhanced inhibitory effects on GBM-induced angiogenesis and cell viability, thereby stressing the importance of further evaluation of this targeting approach in a clinical setting.

**Electronic supplementary material:**

The online version of this article (doi:10.1186/s12935-016-0309-2) contains supplementary material, which is available to authorized users.

## Background

Glioblastoma (GBM) is a devastating tumor of the brain and current therapies have only a palliative effect. GBM tumors are proliferative and infiltrative with a prominent angiogenic phenotype [1]. Thus, therapies targeting angiogenesis have become interesting in the treatment of GBM and the humanized antibody bevacizumab targeting vascular endothelial growth factor A (VEGF) are approved for patients with recurrent GBM [2]. However, as the effect of this and other anti-angiogenic therapies tested in GBM are very limited [3], new alternative strategies for targeting GBM in general and angiogenesis in particular are needed.

GBM is often associated with mutation and amplification of the epidermal growth factor receptor (EGFR) gene and consequently, the importance of EGFR signaling for tumor development and maintenance has gained much attention [4]. Overexpression of EGFR has been correlated to the malignant phenotype of GBM and the most common EGFR mutation in GBM EGFRvIII leads to constitutive active signaling [5–8]. Activation of EGFR downstream signaling pathways leads to increased proliferation and tumorigenesis, and stimulates angiogenesis via up-regulation of pro-angiogenic molecules in the tumor cells [9, 10]. In line with this finding, anti-EGFR therapies have been shown to reduce the production of the pro-angiogenic factor VEGF and reduce vascular formation [11, 12]. Similarly to EGFR, the Notch pathway has also gained attention as a potential target in GBM. The notch gene family consists of four transmembrane receptors (notch1–4) and their ligands (jagged1–2 and Dll1, Dll2 and Dll4) [13]. Ligand binding to the receptor results in two successive proteolytic cleavages which activate downstream signaling resulting in transcription of downstream targets such as Hes1 and Hey1 [14]. The Notch pathway has been linked to a number of GBM specific processes including cellular responses to hypoxia, angiogenesis and tumor growth [15, 16]. Thus, the Notch pathway represents a highly interesting therapeutic target.

Increasing evidence points to a cross-talk between the Notch and EGFR pathway [17, 18]. In line with this, GBM tumors with EGFR amplification display overexpression of notch-regulated genes [19] and it has been shown that notch signaling can induce EGFR upregulation through a P53-dependent mechanism in GBM [20]. It is also believed that the interplay between notch and EGFR is involved in the genesis and maintenance of tumor cells in various cancers including GBM [18, 21]. Thus, this study aimed at investigating the functional interplay between EGFR and notch signaling and elucidating its role in GBM cell maintenance and GBM-induced endothelial cell (EC) sprouting as a surrogate marker for angiogenesis-like processes.

This was done by evaluating the effect of mono- or combined therapy using the tyrosine-kinase inhibitor iressa (TKI; targeting EGFR) and the gamma-secretase inhibitor DAPT (GSI; targeting notch signaling). In the present study, we have used two primary GBM cell cultures with confirmed notch and EGFR expression. Both iressa as well as DAPT single-agent treatment abrogated EGFR and notch signaling, respectively, leading to reduced cell viability, and decreased VEGF expression and GBM-induced EC sprouting. Upon combinational treatment with both iressa and DAPT, the inhibitory effect on cell viability and EC sprouting was even more pronounced. Our data indicate that the cross-talk between EGFR and Notch signaling pathways are crucial for GBM maintenance and vascular phenotype.

## Methods

### Cell cultures

GBM cell cultures used in this study were CPH036 (p6) and CPH047 (p3m1). These were established from patient tumor tissue derived from initial surgery before any other treatment and have previously been described in regard to EGFR status and expression of markers related to stemness and the neuronal lineages [22]. We further analyzed the IDH status of the cell cultures by dideoxy sequencing of *IDH1* codon 132 and *IDH2* codon 140 and 172. Both cell lines were found to be *IDH1/2* wild-type (unmutated). Cells were cultured as floating neurospheres in Neurobasal^®^-A media (NB media) supplemented with N2, B27, bFGF (10 ng/ml), EGF (10 ng/ml), l-glutamine, penicillin (50 U/ml), and streptomycin (50 µg/ml) (all from Invitrogen) and incubated in cell culture flasks (NUNC) in a humidified chamber with 5 % CO_2_ at 37 °C. Spheres were dissociated at every experiment and at new passage to obtain single cells. Endothelial cells (EC) used in this study represents primary human dermal microvascular endothelial cells (HMVEC) from Lonza. EC were incubated in endothelial growth medium-2 (EGM-2) added EGM-2 microvascular (MV) supplements (VEGF, EGF, bFGF, long R3 insulin-like growth factor (R^3^-IGF-1), ascorbic acid, hydrocortisone, GA-1000 and 5 % fetal calf serum (FCS); all from Lonza. Cells were incubated at 5 % CO_2_ at 37 °C and passaged at sub-confluence.

### Reagents

Drugs used in experiments were DAPT (*N*-[(3,5-difluorophenyl)acetyl]-L-alanyl-2-phenyl]glycine-1,1-dimethylethyl ester) obtained from Merck Millipore and iressa (Gefitinib) from tocris bioscience. All drugs were dissolved in DMSO which was also used for treatment controls. Recombinant human VEGF_165_ from Miltenyi Biotec was used to induce a pro-angiogenic response.

### Western blotting

Protein lysates for western blotting (WB) were prepared and obtained from cell pellets by sonication in ice-cold modified RIPA buffer [50 mM Tris–HCl (pH 7.4), 1 % NP40, 0.25 % Na-deoxycholate, 150 mM NaCl, 1 mM EDTA] supplemented with protease and phosphatase inhibitor mixture II and III (calbiochem). Determination of protein concentrations was done by the BCA protein assay (pierce). WB was performed by separation of protein lysates on NuPage 4–12 % Bis–Tris gels following electroblotting onto nitrocellulose membranes using the Novex NuPAGE SDS-PAGE gel system (invitrogen). Membranes were blocked in 5 % non-fat dry-milk in wash buffer for one hour at room temperature following incubation with primary antibodies overnight at 4 °C. Primary antibodies used are displayed in Additional file 1: Figure S4. The following day membranes were washed and incubated with HRP-conjugated secondary antibodies for 1 h at room temperature and developed using the SuperSignal West Dura Extended Duration Substrate (pierce biotechnology) and the biospectrum imaging system (UVP).

### Quantitative real-time PCR (Q-RT-PCR)

Total RNA was purified from GBM cell pellets as previously described [23]. In short, RNA was obtained by using the RNeasy Mini kit and QIAshredder and submitted to a DNase treatment (all from Qiagen). For cDNA synthesis and Q-RT-PCR reactions the SuperscriptTM III platinum^®^ two step qRT-PCR kit with SYBR^®^ Green (Invitrogen) was used. Gene expression levels were quantified according to the comparative Ct method and normalized to expression of the three housekeeping genes TOP1, EIF4A2, and CYC1 (primerdesign). Primers used in Q-RT-PCR reactions for amplification of target genes are displayed in Additional file 1: Figure S5.

### Cell viability assay

Cells were plated at concentrations of 2.5–3.5 × 10^4^ cells per well in 96-well plates and incubated for 7 days with either 100 µl of growth medium or medium containing indicated treatments or control. Cell viability was measured using a 3-(4,5-dimethylthiazol- 2-yl)-2,5-diphenyl-tetrazolium bromide (MTT) assay (sigma) by the addition of 20 µl of MTT solution (5 mg/ml, dissolved in sterile water) to each well and incubation for 4 h before the addition of 100 µl of solubilization buffer (10 % sodium dodecyl sulfate, 0.01 M HCl). Absorbance at 570 nm was measured the next day using Synergy2 microplate reader with Gen5 software.

### VEGF enzyme-linked immunosorbent assay (ELISA)

One million cells were grown for 14 days in 10 ml of culture media added vehicle or inhibitors for 14 days. Conditioned media was collected and VEGF (-A) levels were quantified using the Human VEGF Quantikine ELISA kit (R&D Systems) following manufacturer’s instructions. Quantification was done by measuring the absorbance at 450 nm with 570 as a reference using the Synergy2 microplate reader.

### Spheroid sprouting assay

A spheroid sprouting assay was employed in order to assess the angiogenic-like sprouting process in response to pro-angiogenic stimulus and principally performed as described previously [24]. Cell spheroid formation was obtained by seeding 2000 HMVEC (EC) in each well in non-adherent round-bottomed 96-well plates in growth medium (EGM-2MV) containing 0.3 % methylcellulose (Sigma-Aldrich) and incubated at 37 °C and 5 % CO_2_ for 24 h. Next, the single spheroids from the wells were collected and embedded into collagen gels, consisting of a collagen solution (1 mg/ml rat tail collagen from BD Biosciences) with 0.2 M NaOH, 1 × Medium 199 (Sigma-Aldrich), 0.6 % methylcellulose, (Sigma-Aldrich) in HMVEC basal medium (EBM-2), in 4-well plates. For each well containing spheres, these were stimulated with 50 % FCS in EBM-2 medium added the experimental factor. In the cases where this was conditioned media from GBM cells, the media was collected from 1 × 10^6^ cells grown for 14 days with or without the indicated treatments and subsequently up-concentrated around 10 times by centrifugation at max speed using Amicon Ultra Centrifugal Filter Units (Merck Millipore). Spheres were incubated for 16 h and spheroid sprouting was visualized by using an Eclipse TS100 phase-contrast microscope, Digital Sight imaging system and the NIS Elements F3.2 software (all from Nikon). Quantification of spheroid sprouting (number of sprouts and total sprout length per sphere) was determined using ImageJ software.

### Statistics

Statistics were performed using a one-way Analysis of Variance test (ANOVA) to compare multiple data groups, followed by Tukeys post hoc test, for comparison of multiple samples or by an un-paired two-tailed student’s t test when comparing two samples. The software used for the above statistics and creation of figures was Graphpad Prism 6.0. The effect of combination therapy was done in SAS version 9.4 (SAS Institute, Denmark) by general linear modeling and analysis of the response levels was done on the log scale. Tests for additivity were made by comparing the sum of the two treatment effects on the log scale with the combination treatment and the hypothesis of additive effect were rejected if the comparison demonstrated significant interaction i.e. evidence of synergistic or sub-additive effect. A *p* value < 0.05 was considered statistically significant.

## Results

### Characterization of GBM cell cultures for EGFR- and notch signaling pathway component expression

**Fig. 1:**
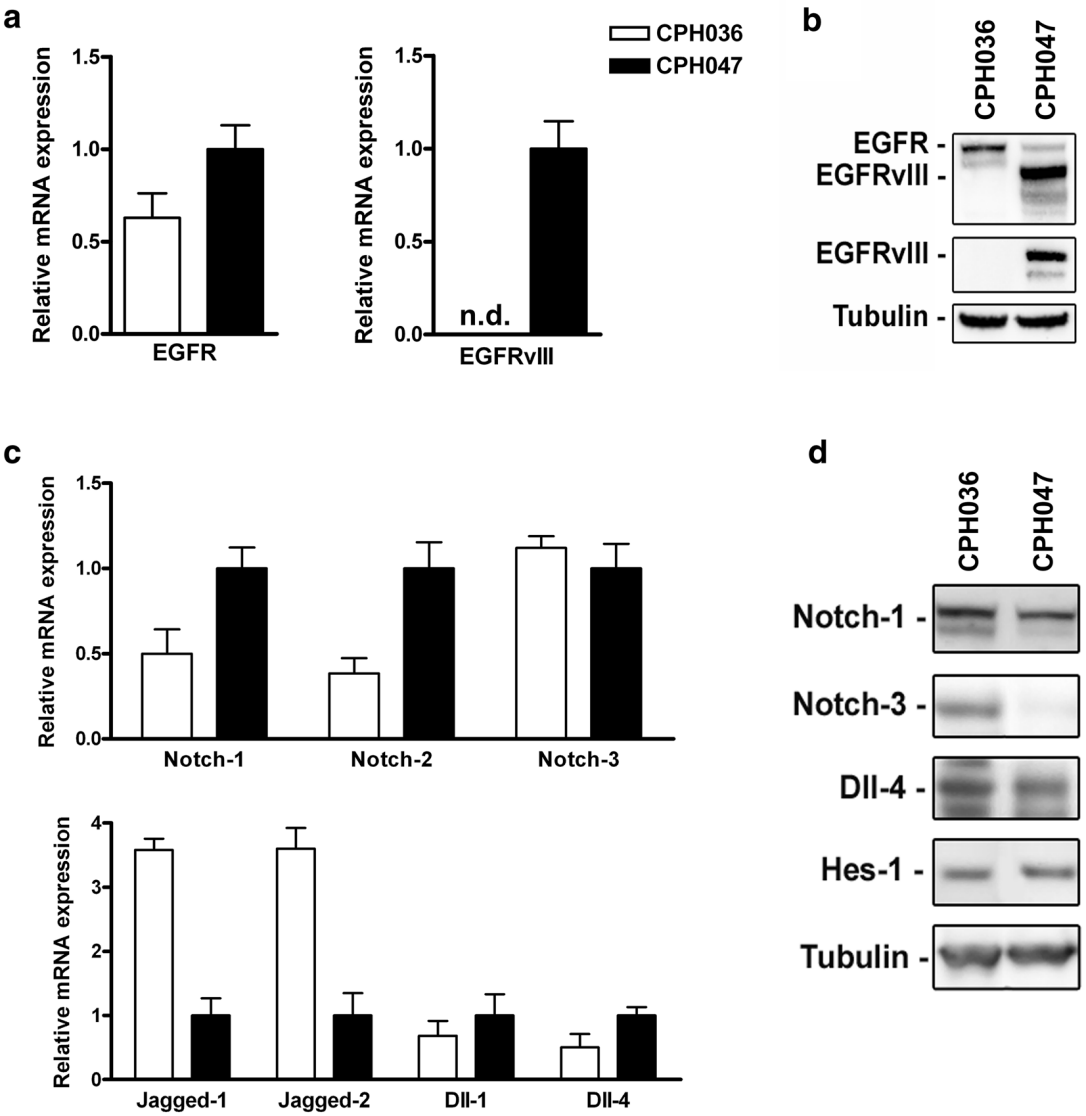
Expression levels of EGFR and notch molecules in two primary GBM cell cultures (CPH036 and CPH047). **a**, **c** Q-RT-PCR detection of EGFR, EGFRvIII, notch 1–3, jagged 1–2, Dll-1 and Dll-4 mRNA expression in CPH036 and CPH047 cells. EGFRvIII was not detected (ND) in CPH036 cells (mean ± SEM, n = 3). **b**, **d** WB analysis of EGFR, EGFRvIII, notch 1- and -3, Dll-4 and Hes-1 protein in CPH036 and CPH047 cells. Tubulin serves as loading control

Q-RT-PCR and western blotting (WB) were employed in order to determine the expression levels of EGFR and EGFRvIII and Notch family molecules in two primary GBM cell cultures (CPH036 and CPH047). Both mRNA and protein analysis found that the two cultures were positive for EGFR, whereas only CPH047 displayed expression of the mutated EGFR variant, EGFRvIII, (Fig. 1a, b). Further, mRNA analysis found that both cell cultures express notch receptors 1–3 and their notch receptor ligands jagged-1, jagged-2, Dll-1 and Dll-4 (Fig. 1c) and the expression was confirmed when examining a selection of these molecules at the protein level (Fig. 1d). Protein expression of the notch downstream effector protein, Hes-1, confirmed active Notch signaling in both cultures (Fig. 1d).

### Iressa and DAPT abrogates downstream survival pathway signaling through the EGFR- and notch pathways and reduces cell viability in vitro

**Fig. 2:**
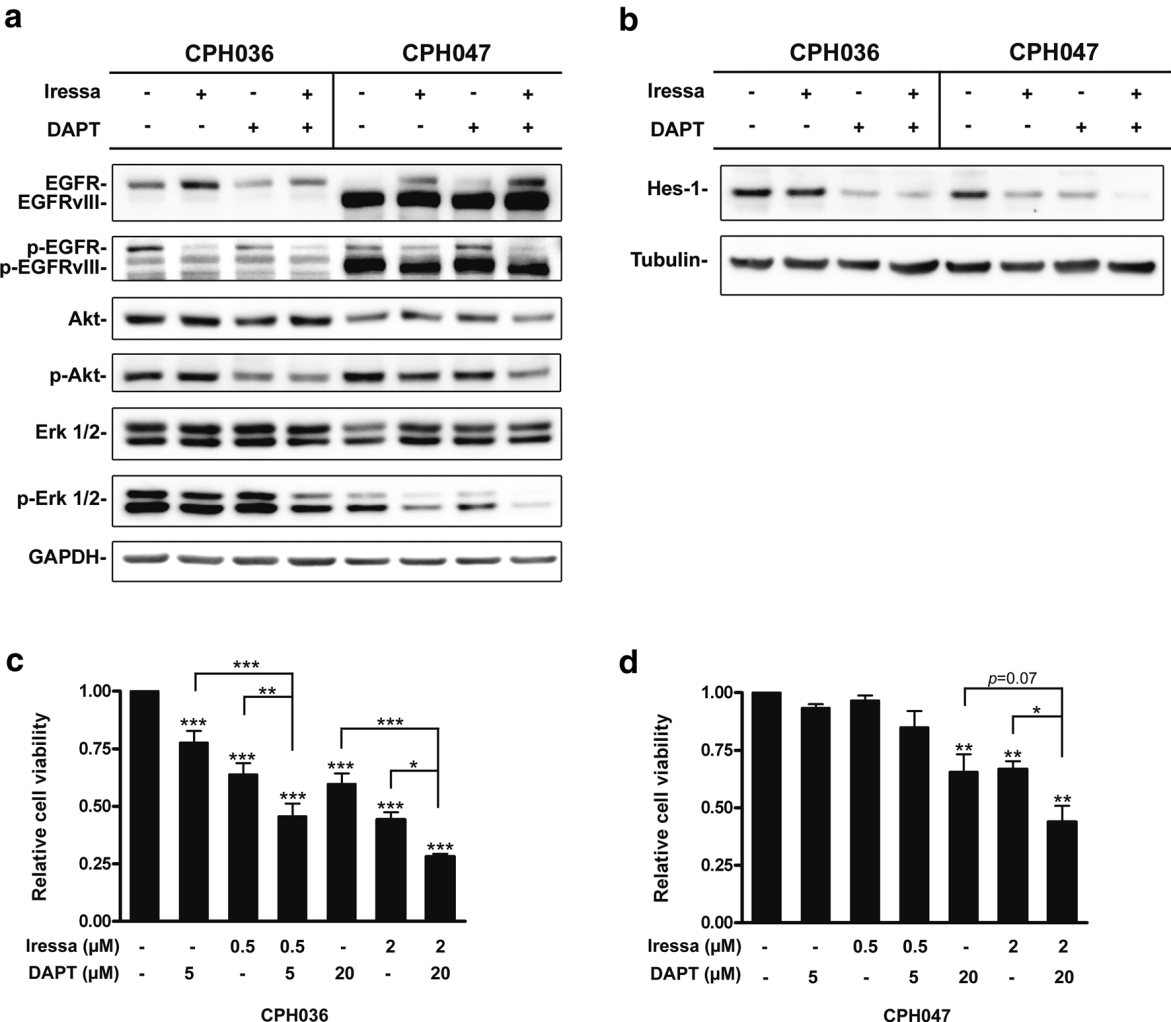
Iressa and DAPT treatment inhibits the EGFR- and notch pathways abrogating downstream signaling and cell viability in vitro. **a**, **b** WB analysis of CPH36 and CPH047 cells treated with 5 µM iressa, 5 µM DAPT or a combination for 24 h. WB was investigated for expression of EGFR, EGFRvIII, Akt, p-Akt, Erk, p-Erk and Hes-1. GAPDH and tubulin serves as loading controls. **c**, **d** Cell viability of CPH036 or CPH047 cells following 7 days of treatment with iressa, DAPT or a combination in the indicated doses (mean ± SEM, n = 5). *p < 0.05, **p < 0.01, ***p < 0.001

Following verification that the GBM cells expressed components of the EGFR- and notch signaling pathways, we wanted to investigate the effect of EGFR and Notch inhibition. We used the EGFR inhibitor iressa, and the notch inhibitor DAPT for investigating the effect of EGFR and notch signaling abrogation on the downstream survival kinases Akt and Erk. In CPH036 cells, mono-therapy with iressa (5 µM) inhibited EGFR phosphorylation (pY1086) but had no effect on phosphorylation of the downstream effector proteins Akt (p-Akt) and Erk (p-Erk) as seen in Fig. 2a. DAPT (5 µM) mono-therapy had minor effect on p-EGFR and displayed inhibition of p-Akt but without effect on p-Erk. Upon combined Iressa and DAPT treatment this resulted in both inhibition of p-Akt and p-Erk in CPH036 cells. In CPH047 cells mono-therapy with either Iressa or DAPT reduced p-Akt and p-Erk levels to some degree and upon combined treatment this effect was even more pronounced (Fig. 2a). Furthermore, as seen in Fig. 2b, mono-therapy, with Iressa or DAPT, decreased Hes-1 expression in CPH047 cells whereas only DAPT could inhibit Hes-1 expression in CPH036 cells. Upon combinational treatment with Iressa and DAPT an additive downregulation of Hes-1 expression was seen in CPH047 cells, whereas no direct additive effect could be seen in the CPH036 cells.

Following confirmation that the inhibitors abrogated downstream signaling through survival pathways Akt and Erk, we examined the effect of Iressa and DAPT on cell viability in vitro. As seen in Fig. 2c single-agent treatment of the CPH036 cells, with either DAPT or Iressa, was able to significantly decrease cell viability compared to control. Upon co-administration of both drugs, this effect was even further potentiated and confirmed to be additive, as a test for additivity was not rejected (*p* = 0.56 for 0.5 μM iressa + 5 μM DAPT and *p* = 0.50 for 2 μM Iressa + 20 μM DAPT). In CPH047 cells, higher concentrations (20 µM DAPT or 2 µM iressa) of each inhibitor were needed to significantly inhibit cell viability and upon combined treatment this inhibitory effect was further enhanced and again confirmed to be additive (*p* = 0.98) (Fig. 2d). In conclusion, combinational therapy with Iressa and DAPT display pronounced inhibitory effect as compared to mono-therapy in GBM cells on both downstream signaling of the EGFR- and notch pathway and cell viability.

### Capacity of GBM cell cultures to secrete and express VEGF and to induce endothelial cell sprouting

**Fig. 3:**
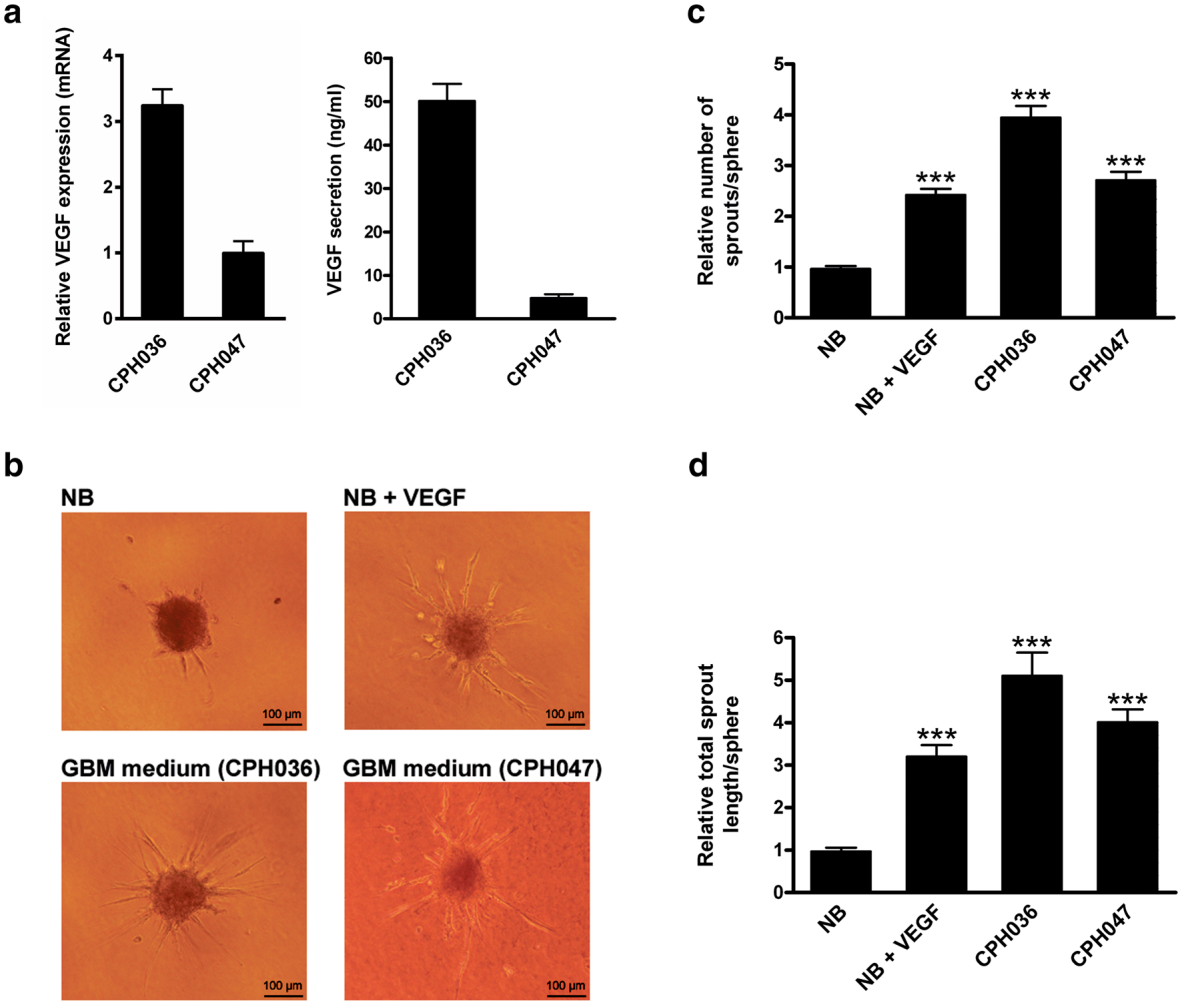
EC cell sprouting stimulation by GBM-conditioned cell culture media. CPH036 and CPH047 cells were grown for 14 days and the conditioned media was collected and concentrated. EC were grown in presence of 20 ng/ml VEGF or conditioned media from CPH036 or CPH047 cells. **a** Relative VEGF expression analyzed by Q-RT-PCR, and VEGF secretion (ng/ml) analyzed by ELISA of CPH036 and CPH047 cells (mean ± SEM, n = 6 for Q-RT-PCR and n = 3 for ELISA). **b** Representative pictures showing sprouting of EC upon stimulation with VEGF or conditioned media from either CPH036 or CPH047 cells. **c**, **d** EC sprouting quantified as number of sprouts and total length of sprouts per sphere, respectively, and displayed relative to NB unconditioned media (mean ± SEM, n = 3 measuring an average of 10 spheres per condition in each experiment). *Scale bar* shows 100 μm. ***p < 0.001 vs. NB medium

VEGF is a well-known inducer of angiogenesis in GBM [25]. This prompted us to investigate the level of VEGF expression and secretion in CPH036 and CPH047 cells. We found that both cell cultures were positive for VEGF mRNA expression and protein secretion (Fig. 3a) together with other pro-angiogenic factors (Additional file 1: Figure S1). The expression of VEGF receptors (VEGFR-1 and -2) could not be detected in the GBM cells (Additional file 1: Figure S2), suggesting only paracrine effects of VEGF upon secretion from the tumor cells. Knowing that the GBM cells secrete VEGF into the culture media we assessed whether conditioned media from CPH036 and CPH047 was sufficient in inducing angiogenic-like processes in EC. We performed a cell sprouting assay which measures the cells ability to migrate, proliferate and form tube-like structures, all processes required in angiogenesis. Upon exposure of EC to conditioned media obtained from either CPH036 or CPH047 cells this clearly induced sprouting as displayed in Fig. 3b. The relative number of sprouts per sphere and relative total sprout length per sphere was quantified to be significantly increased compared to control (NB unconditioned media) as shown in Fig. 3c, d. These data implied that the examined GBM cells have the capacity of inducing angiogenesis-like processes of EC in vitro possibly through secretion of VEGF.

### Iressa and DAPT abrogates GBM-induced endothelial cell sprouting and reduces VEGF expression and secretion by GBM cells

**Fig. 4:**
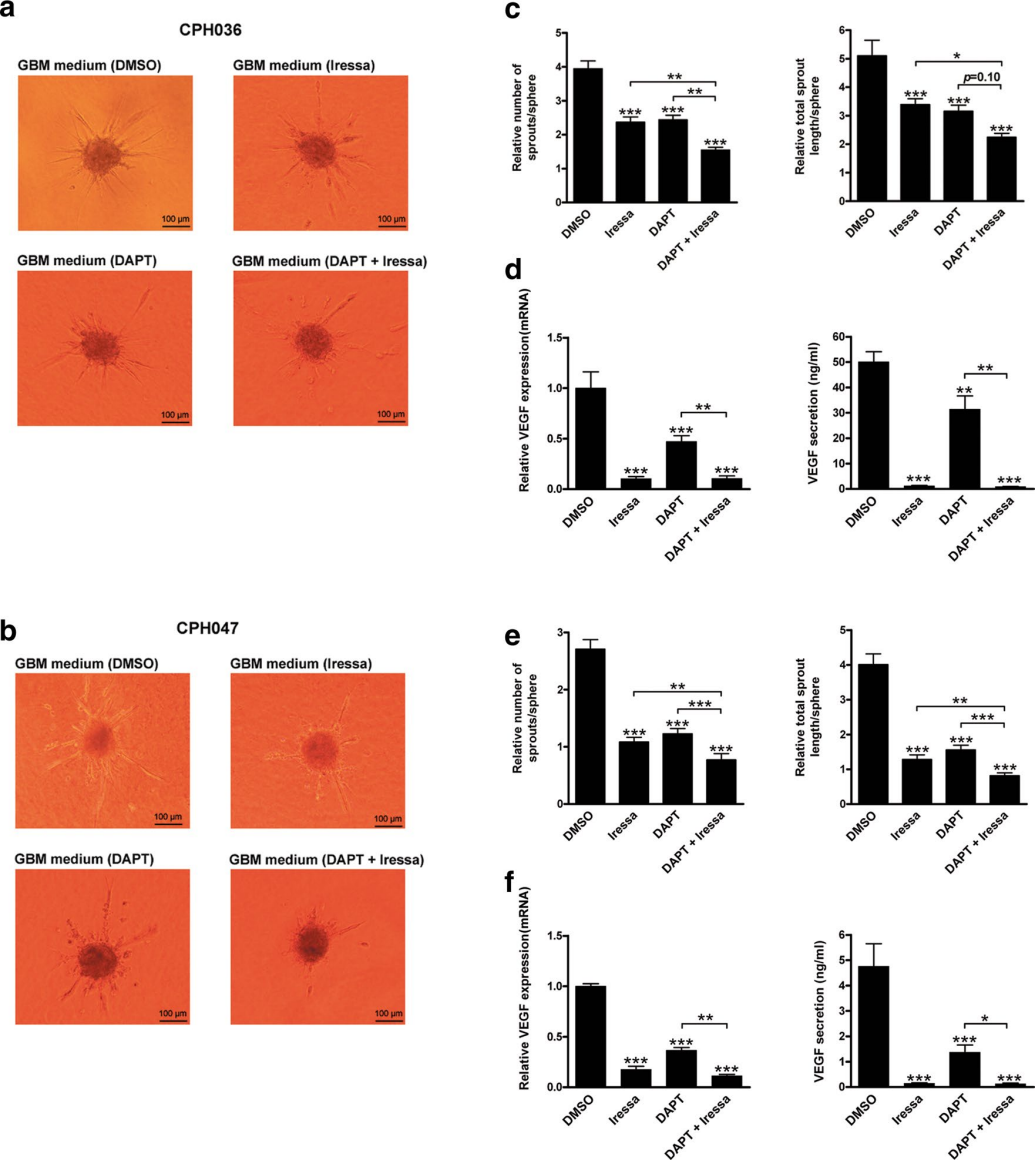
Iressa and DAPT treatment abrogates GBM-induced EC sprouting, and inhibits VEGF expression and secretion in GBM cells. CPH036 and CPH047 cells were treated with 5 µM iressa, 5 µM DAPT or a combination for 14 days. The conditioned GBM media was concentrated and subjected to EC for sprouting analysis. **a**, **b** Representative pictures showing sprouting of EC upon administration of conditioned media obtained from CPH036 or CPH047 cells treated with indicated inhibitors. **c**, **e** Relative EC sprouting quantified as total number of sprouts and total length of sprouts per sphere upon stimulation with conditioned inhibitor-treated media from either CPH036 or CPH047 cells, respectively (mean ± SEM, n = 3 measuring an average of 10 spheres per condition in each experiment). **d**, **f** VEGF mRNA levels in inhibitor-treated CPH036 and CPH047 cells were analyzed by Q-RT-PCR and normalized to the mean gene expression of the control. VEGF secretion levels (ng/ml) analyzed by ELISA from inhibitor-treated CPH036 and CPH047 cells as measured by VEGF-ELISA (mean ± SEM, n = 3). *Scale bar* shows 100 μm. *p < 0.05, **p < 0.01, ***p < 0.001

Following confirmation that the GBM cells displayed capacity to induce EC sprouting, we investigated how this ability was affected by EGFR and Notch inhibition. EC were subjected to conditioned media collected from GBM cells receiving either no treatment (DMSO) or treatment with 5 µM iressa and 5 µM DAPT alone or in combination (5 µM DAPT +5 µM iressa). We found that mono-therapy with Iressa or DAPT of CPH036 and CPH047 cells significantly reduced the capacity of GBM-induced EC spheroid sprouting (Fig. 4a–c, e). Upon treatment with combined DAPT and iressa an increased co-inhibitory effect of quantified EC sprouting could be seen compared to mono-therapy (Fig. 4c, e). In CPH036 cells, the co-inhibitory effect was confirmed to be additive for both the number (*p* = 0.62) and length (*p* = 0.59) of spouts. For the CPH047 cells the length of spouts was borderline non-significant (*p* = 0.080), demonstrating a trend towards additivity. Conversely, the co-inhibitory effect for the number of sprouts in CPH047 cells showed a significant interaction (*p* = 0.046), but with an effect that was less (76 % reduction) than would be expected if additive (83 % reduction), suggesting that the combination was sub-additive. Further, the inhibitory effect of DAPT and Iressa on EC sprouting could be confirmed not to be a result of non-metabolized inhibitor leftovers inducing EC death since conditioned media from GBM cells treated with DAPT, Iressa or a combination had no effect on EC proliferation (Additional file 1: Figure S3). To examine whether the effect of inhibited GBM-induced EC sprouting could be due to the effect of Iressa and DAPT on VEGF expression and secretion by the GBM cells, we measured VEGF secretion following treatment. Iressa treatment resulted in almost complete inhibition of VEGF expression and secretion in CPH036 and CPH047 cells (Fig. 4d, f), while DAPT treatment was able to partially abrogate VEGF expression and secretion, however with less potency, compared to iressa. Upon combined treatment no additive effect could be observed as a result of almost complete inhibition of VEGF secretion and expression by Iressa treatment (Fig. 4d, f). Summed, both iressa and DAPT display capacity to inhibit GBM-induced cell sprouting in EC and upon combinational treatment this effect is even further enhanced. Further, the results indicate that this effect, at least partly, could be a result of inhibition of VEGF expression.

## Discussion

EGFR and notch are both involved in regulation of GBM cancer cells by promoting their survival, therapeutic resistance and pro-angiogenic signaling [13, 26, 27]. Thus, there is a rationale for treatment with inhibitors targeting both the EGFR and Notch signaling axis in GBM. The main focus of this study was to investigate the effect of simultaneous EGFR and notch abrogation on GBM cell maintenance and EC sprouting.

Aberrant expression of components of the EGFR and notch pathway has in GBM been confirmed previously [27, 28]. In accordance with this, we identified heterogeneous expression of EGFR/EGFRvIII, notch ligands and notch receptors in our GBM cell cultures. EGFR and Notch are important regulators of angiogenesis and abrogation of either of these pathways results in reduced angiogenesis in GBM [15, 29]. In the examined GBM cells, we confirmed endogenous expression and secretion of the key pro-angiogenic cytokine VEGF [25], for which increased expression has been correlated with increased glioma malignancy and poor prognosis [30, 31]. Furthermore, we confirmed that the examined GBM cells were able to induce EC sprouting (Fig. 3b–d) indicating that these cells had the capacity to induce neo-angiogenesis of surrounding EC by the secretion of pro-angiogenic factors.

Upon abrogation of Notch and EGFR signaling by DAPT or Iressa treatment, respectively, this inhibited the expression and secretion of VEGF in our GBM cells (Fig. 4d, f). This supports that VEGF-induced angiogenesis is dependent of active signaling through the notch and EGFR pathways as also shown by others [32–34]. Recently, Wang et al. [29] showed that combined treatment with the anti-EGFR antibody Cetuximab together with DAPT displayed downregulation of VEGF in Head Neck Squamous Cell Carcinoma [29], which is in line with our observations. VEGF is generally considered to be a positive upstream regulator of Notch with Notch acting as an upstream regulator of VEGFRs [35]. Moreover, our data demonstrate that Notch regulates VEGF expression, indicating the existence of a positive feedback-loop regulatory mechanism.

Studies have shown that treatment with small molecule inhibitors targeting EGFR or Notch is able to inhibit GBM angiogenesis in vitro [36, 37]. We observed that treatment with DAPT plus iressa was not sufficient to fully block EC sprouting (Fig. 4c, e) despite almost complete inhibition of VEGF secretion upon combined treatment (Fig. 4d, f) suggesting that other angiogenic stimulators are involved in GBM-induced EC sprouting. Factors including angiogenin, PDGF-AA, IGFBP-3 are known to be implicated in angiogenesis [38–40] and were confirmed to be present in the GBM-conditioned media at comparable levels to VEGF (Additional file 1: Figure S1) which could explain additional stimulation of EC sprouting.

Aberrant EGFR and notch signaling regulate cell viability and therapeutic resistance of GBM cells [17, 27, 41]. Both, EGFR and notch regulated signaling are in GBM linked to the RAS-RAF-MEK-ERK and the PI3K-Akt-mTOR signaling pathways [42, 43]. Interestingly, it has been shown that Notch signaling is dependent on mTOR in lung and kidney tumor cells [44], indicating the existence of a positive feedback loop between Notch and EGFR signaling. Our results show that the inhibition of EGFR signaling results in decreased Hes-1 levels supporting that EGFR signaling stimulate activity of the Notch pathway. Further, we found upon combined treatment targeting both Notch and EGFR an increased inhibition of GBM cell viability compared to mono-therapy alone. This was probably a result of more effective inhibition of the pro-survival pathways Akt and Erk which we observed upon combination therapy (Fig. 2a). Cenciarelli et al. [28] showed that co-treatment with GSI-X and AG1478 (targeting Notch and EGFR, respectively) displayed synergistic anti-proliferative effects in GBM in vitro [28]. Taken together, our data and those of others indicate redundant signaling between EGFR and Notch, which indicate a need for further preclinical and clinical evaluation of simultaneous inhibition of Notch and EGFR which are upstream of key pro-survival regulatory pathways as the RAS-RAF-MEK-ERK and PI3K-Akt-mTOR.

Over the last years, a number of pharmacological studies have been conducted testing either EGFR or Notch pathway inhibitors in patients with various cancer types including GBM (http://www.clinicaltrial.gov). For GBM patients a number of different EGFR targeting drugs have been tested in the clinic, but overall results have been disappointing with non or very limited clinical benefits [45]. So far, only one study has been reported for the use of a notch-specific inhibitor in glioma patients. In this study, the Merck-developed GSI termed MK-0752 was tested in various advanced solid tumors and the results indicated some clinical benefits especially in glioma patients [46]. Still, the anti-tumor activity was not impressive with most patients obtaining stable disease as best response [46]. Data from a currently ongoing phase II trial, treating patients with recurrent or progressive GBM with another GSI (RO4929097), are yet to be published, but will further shed light on the effect of single-agent treatment with Notch inhibitors. Overall, the results from recent and/or ongoing clinical trials evaluating EGFR- and notch-specific inhibitors as mono-therapies imply certain clinical limitations of this approach.

In this study, we find that a combined treatment strategy that targets both EGFR and notch signaling pathways results in enhanced inhibitory effect on cell viability and EC sprouting, compared to either of the mono-therapies, supporting the important role of notch/EGFR signaling cross-talk in GBM. Taken together, this fact and the above mentioned clinical studies support the rationale for combined treatment strategy employing both EGFR and notch inhibitors.

## Additional file

10.1186/s12935-016-0309-2 Evaluation of angiogenic factors secreted by GBM cells. **Figure S2.** Expression of VEGF receptors (VEGFR-1 and VEGFR-2) in GBM cell cultures CPH036 and CPH047 and in endothelial cells (HMVEC). **Figure S3.** Effect of conditioned cell media from GBM cell cultures treated with inhibitors on endothelial cell proliferation. **Figure S4.** Overview of primary antibodies used for western blotting. **Figure S5.** Overview of primer sets used for Q-RT-PCR.

## Abbreviations

GBM: glioblastoma
EGFR: epidermal growth factor receptor
VEGF: vascular endothelial growth factor A
TKI: tyrosine-kinase inhibitor
GSI: gamma-secretase inhibitor
EC: endothelial cell
