# Supplementary material for: Combined EGFR- and notch inhibition display additive inhibitory effect on glioblastoma cell viability and glioblastoma-induced endothelial cell sprouting in vitro
Source: Cancer Cell Int. 2016 Apr 26;16:34. doi: 10.1186/s12935-016-0309-2 (PMC4845362; doi:10.1186/s12935-016-0309-2)
Supplement: Supplementary file 1 — 10.1186/s12935-016-0309-2 Evaluation of angiogenic factors secreted by GBM cells. Figure S2. Expression of VEGF receptors (VEGFR-1 and VEGFR-2) in GBM cell cultures CPH036 and CPH047 and in endothelial cells (HMVEC). Figure S3. Effect of conditioned cell media from GBM cell cultures treated with inhibitors on endothelial cell proliferation. Figure S4. Overview of primary antibodies used for western blotting. Figure S5. Overview of primer sets used for Q-RT-PCR. [file 12935_2016_309_MOESM1_ESM.pdf]

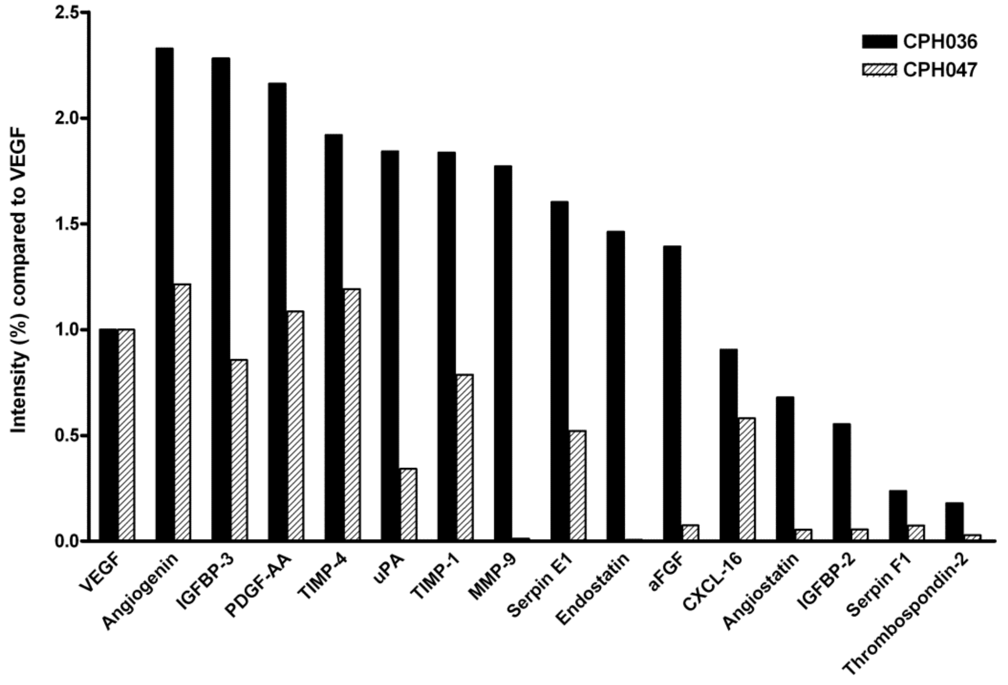

Supplementary Figure 1. Evaluation of angiogenic factors secreted by GBM cells. Relative secretion of angiogenic factors set relative to VEGF secretion. One million GBM cells (CPH036 and CPH047) were incubated for 14 days and the conditioned cell media was harvested, concentrated around 10 times and subjected to the Proteome Profiler human angiogenesis array kit (R&D systems) used in accordance with the manufacturer's instructions. Signal was detected utilizing the Super-Signal West Dura extended Duration Substrate (Pierce Biotechnology) in the UVP Biospectrum AC imaging system which also was used for quantification of relative protein expression. Mean of one experiment performed in duplicate is shown.

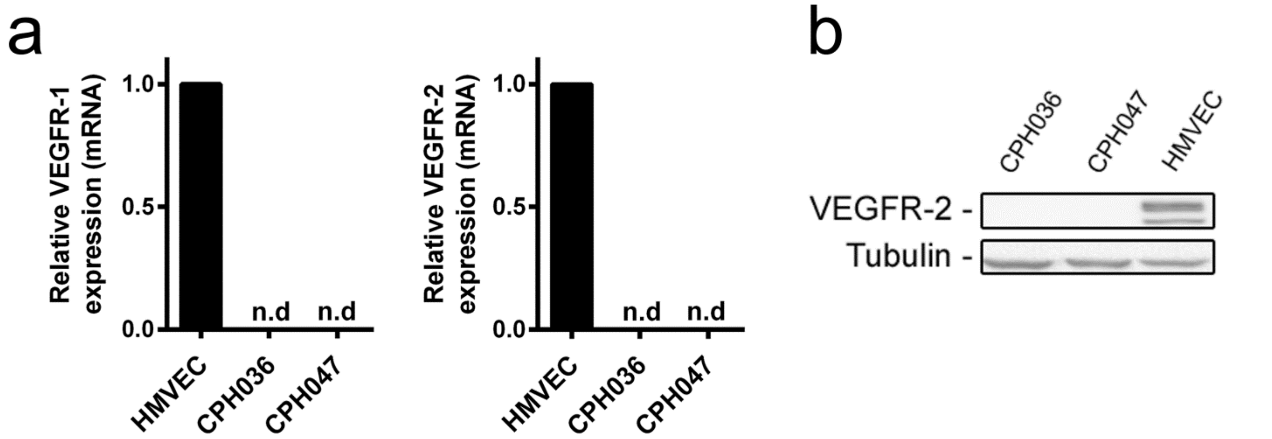

Supplementary Figure 2. Expression of VEGF receptors (VEGFR-1 and VEGFR-2) in GBM cell cultures CPH036 and CPH047 and in endothelial cells (HMVEC). (a) Expression levels of VEGFR-1 and VEGFR-2 evaluated by Q-RT-PCR. (b) Expression of VEGFR-2 identified by western blotting using primary antibody against VEGFR-2 and tubulin.

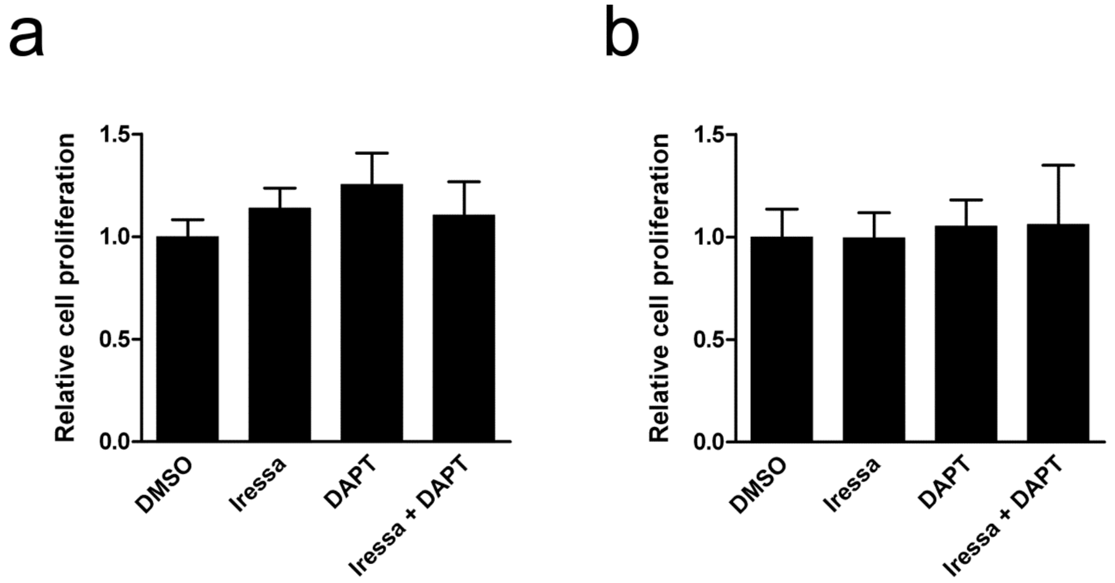

Supplementary Figure 3. Effect of conditioned cell media from GBM cell cultures treated with inhibitors on endothelial cell proliferation. Conditioned media was obtained from $1x10^6$ cells of either CPH036 (a) or CPH047 (b) cells treated for 14 days with DMSO, 5µM Iressa, 5µM DAPT or a combination. HMVEC (EC) cells were plated in 96-well plates (2,000 cells in 0.1 mL EC media) and the following day the media was changed to EC media added 10% of conditioned media from the GBM cells that had been concentrated around 10 times. Cells were incubated for 20 hrs and level of proliferation was examined by BrdU assay using the Cell Proliferation ELISA, BrdU kit (Roche A/S) using a 20 hrs BrdU incubation step and otherwise following the instructions by the manufacturer. Quantification was done by measuring the absorbance at 370nm with 492nm as a reference using Synergy2 microplate reader with Gen5. Data are shown as mean ± SEM obtained from three independent experiments. Significant difference was tested with a one-way ANOVA with a Tukey post-hoc test.

Dilution	Antibody	Manufacturer
1:1000	Rabbit anti-Akt	Cell Signaling, #9272
1:1000	Rabbit anti-pAkt	Cell Signaling, #9271
1:1000	Rabbit anti-Erk 1/2	Cell Signaling, #9102
1:1000	Mouse anti-pErk 1/2	Cell Signaling, #9106
1:20.000	Sheep anti-EGFR	Fitzgerald, #20-ES04
1:1000	Rabbit anti-pEGFR	Invitrogen, #44-790G
1:50	Mouse anti-EGFRvIII	Duke University, L8A4
1:100	Goat anti-Notch 1	Santa Cruz, #SC-23304
1:1000	Rabbit anti-Notch 3	Cell Signaling, #5276
1:1000	Rabbit anti-Dll 4	Abcam, #ab7280
1:2000	Rabbit anti-Hes 1	Toray Industries inc, Japan
1:1000	Rabbit anti-VEGFR-2	Cell signaling, #55b11
1:1000	Rabbit anti-Tubulin	Cell Signaling, #2125

Supplementary Figure 4. Overview of primary antibodies used for western blotting. Antibodies are listed with dilution, manufacturer (university) and catalog number.

Target gene	Forward primer (5' - 3')	Reverse primer (5' - 3')
EGFR	GGC ATA GGA ATT TTC GTA GTA CAT	TCC TTG GGA ATT TGG AAA TT
EGFRvIII	ATG CGA CCC TCC GGG ACG	ATC TGT CAC ATA ATT ACC T
Dll-1	GCC GAC AAG AAT GGC TTC	CCG GCC TTT TTC TTT CAG
Dll-4	GGT CAG ACC TGG TTA TTG G	CGA AAG ACA GAT AGG CTG
Hes-1	AGC GGG CGC AGA TGA C	CGT TCA TGC ACT CGC TGA A
Notch-1	CTT CCC CTA CGG CCG CGA	CAG GTA GAC GAT GGA GCC GCG GA
Notch-2	GCC TGT ATG TGC CCT GTG CAC C	AGC CTC CAT TGC GGT TGG CAC
Notch-3	CTG GCT GAC AGC TCG GTC ACG C	AGT GGC AGT GGC AGC TGC ATA G
Jagged-1	ATG GGG AGT GTG ATA CCA	GAG ACT GGA AGA CCG ACA
Jagged-2	CGG CCA CCT GGA CAA TAA	CAA CCG TCT CCA CCT TGA
VEGF	CCT TGC TGC TCT ACC TCC AC	ATC TGC ATG GTG ATG TTG GA
VEGFR-1	GGC TCT GTG GAA AGT TCA GC	GTG ACC AAC ATG GAG TCG TG
VEGFR-2	GTG ACC AAC ATG GAG TCG TG	TGC TTC ACA GAA GAC CAT GC

Supplementary Figure 5. Overview of primer sets used for Q-RT-PCR. All primers were obtained from DNA Technology A/S.
